# Insights into Antisite Defect Complex Induced High Ferro-Piezoelectric Properties in KNbO_3_ Perovskite: First-Principles Study

**DOI:** 10.3390/ma17143442

**Published:** 2024-07-11

**Authors:** Bei Li, Yilun Zhang, Meng Wang, Xu Zhang, Xiaofeng Zhang, Kai Liu

**Affiliations:** 1 School of Materials Science and Engineering, Wuhan University of Technology, Wuhan 430070, China; libei@whut.edu.cn (B.L.); 317684@whut.edu.cn (Y.Z.); wangmeng10330@whut.edu.cn (M.W.); 2 Research Center for Materials Genome Engineering, Wuhan University of Technology, Wuhan 430070, China; 3 National Engineering Laboratory for Modern Materials Surface Engineering Technology & The Key Lab of Guangdong for Modern Surface Engineering Technology, Institute of New Materials, Guangdong Academy of Science, Guangzhou 510650, China; zxf200808@126.com

**Keywords:** antisite defects, formation energy, band structure, spontaneous polarization, piezoelectric tensor

## Abstract

Improving ferro-piezoelectric properties of niobate-based perovskites is highly desirable for developing eco-friendly high-performance sensors and actuators. Although electro-strain coupling is usually obtained by constructing multiphase boundaries via complex chemical compositions, defect engineering can also create opportunities for novel property and functionality advancements. In this work, a representative tetragonal niobate-based perovskite, i.e., KNbO_3_, is studied by using first-principles calculations. Two intrinsic types of Nb antisite defect complexes are selected to mimic alkali-deficiency induced excess Nb antisites in experiments. The formation energy, electronic profiles, polarization, and piezoelectric constants are systematically analyzed. It is shown that the structural distortion and chemical heterogeneity around the energetically favorable antisite pair defects, i.e., (${\mathrm{Nb}}_{K}^{4\cdot }+K_{\mathrm{Nb}}^{4\prime }$), lower the crystal symmetry of KNbO_3_ from tetragonal to triclinic phase, and facilitate polarization emergence and reorientation to substantially enhance intrinsic ferro-piezoelectricity (i.e., spontaneous polarization *P*_s_ of 68.2 μC/cm^2^ and piezoelectric strain constant *d*_33_ of 228.3 pC/N) without complicated doping and alloying.

## 1. Introduction

Ferroelectric perovskites are an imperative class of advanced functional materials due to their high light adsorption, high permittivity, switchable polarization, excellent piezoelectricity, pyroelectricity, etc. [1,2,3,4,5]. Among them, niobate-based perovskites have elicited considerable attention in numerous fields. For instance, NaNbO_3_- and AgNbO_3_-based ceramics have been used in energy storage and conversion applications [6,7,8,9,10]. As another typical ferroelectric perovskite, KNbO_3_ is a promising candidate for piezoelectric, photovoltaic, photocatalytic, and moisture-sensitive sensors [11,12,13,14,15]. Although pristine niobate-based perovskites usually suffer from inferior piezoelectric performance as compared with lead zirconate titanate (Pb(Zr,Ti)O_3_), enormous efforts have been dedicated to strengthen strain piezoelectric properties via tailoring multiphase boundaries near room temperature tuned by chemical modification [16,17,18]. The coexistence of distinct phases, i.e., to form morphotropic or polymorphic phase boundaries as in (K, Na)NbO_3_-based ceramics, could lower the energy differences with flattened free energy profiles and consequently facilitates polarization switching and piezoelectric enhancement [19,20,21]. However, the routine strategy of creating composition-induced multiphase boundaries generally involves complex chemical compositions and exhibits intrinsically poor temperature stability, thereby severely hindering commercialized applications.

Meanwhile, structural point defects, i.e., vacancies, dopants, and interstitials, are decisively important for electronic and electrical functionalities of ferroelectric perovskites. In particular, defect complexes, consisting of clusters or aggregates of point defects, play a vital role in ferro-piezoelectric, electro-strain, and electromechanical properties of ferroelectric crystalline materials. For instance, large electro-strain has been generally observed in various lead-free ferroelectric perovskites, which stems from a reversible domain-switching mechanism as a result of energetically favorably formed defect complexes involving vacancies and acceptor/donor dopants on either A- or B-site cations [22,23,24,25]. Furthermore, the coupling between charged hetero-valence dopants and vacancies can generate electric and elastic dipole moments that significantly influence the polarization switching and domain wall motion, thereby giving rise to ferroelectric “hardening” or “softening” depending on the type of doping (i.e., acceptor or donor dopants) [26,27,28]. Whereas these dopants are usually exotic substituents or impurity atoms, antisite point defects and associated defect complexes can be intrinsically formed due to deviations from ideal stoichiometry in chemically simple, lead-free ferroelectrics. Recently, a giant effective piezoelectric coefficient ($d_{33}^{*}$) with a high Curie temperature has been achieved in sputtering deposited alkali-deficient niobate-based perovskite (NaNbO_3_ and (K, Na)NbO_3_) epitaxial thin films, which contain self-assembled antisite or planar defect complexes [29,30,31,32,33]. The formation of excess Nb antisites, accompanied with compensated A-site vacancies or antisites, becomes energetically favorable in these tetragonal niobate perovskites, and their assembly can result in a variety of strain and polar states (i.e., antisite nanopillars and planar faults) together with chemical and structural heterogeneity, which are responsible for the superior electromechanical response. However, a complete and direct picture for underlying principles and mechanisms in relation to the atomic structure of antisite defect complexes is still an imperative requirement, so that these defects can be deliberately tuned and perovskite crystals with improved ferro-piezoelectric properties can be rationally designed.

Undoubtedly, various theoretical investigations of niobate-based perovskites have been performed using first-principles calculations, but to our knowledge, these studies have mostly focused on isolated point defects [34,35,36,37]. Nevertheless, the effects of structural defects on macroscopic ferro-piezoelectric performance should originate from the formation of intricate defect complexes, rather than point defects separately. Therefore, we performed first-principles density functional theory (DFT) calculations of a representative tetragonal niobate-based ferroelectric perovskite model, i.e., KNbO_3_. Two main types of neutral-charged antisite defect complexes, namely, (${\mathrm{Nb}}_{K}^{4\cdot }+4V_{K}^{\prime }$) and (${\mathrm{Nb}}_{K}^{4\cdot }+K_{\mathrm{Nb}}^{4\prime }$) in Kröger–Vink notation, were simulated. The effects of the antisite defects on the structural, electronic, ferroelectric, and piezoelectric properties of KNbO_3_ single crystals were also thoroughly examined. The ultimate objective of this work is thus to fill the knowledge gap and provide informative and comprehensive understanding of the underlying physical mechanisms of antisite defect complexes induced superior ferro-piezoelectricity in niobate-based perovskites from an electronic/atomic perspective.

## 2. Computational and Structural Details

In this work, all first-principles DFT calculations are carried out using the Vienna ab-initio simulation package (VASP) through a plane-wave basis set with the projector augmented wave (PAW) method for the core-valence interactions [38,39,40]. The exchange-correlation energy of electrons is depicted by generalized gradient approximations (GGAs) in the form of the Perdew–Burke–Emzerh (PBE) scheme [41,42]. K 3s^2^3p^6^4s^1^, Nb 4p^6^5s^1^4d^4^, and O 2s^2^2p^4^ are treated as semi-core states, and are included in the valence. The energy cutoff for the plane-wave basis set is 600 eV. The Brillouin zone integrations are sampled by using a gamma-centered method with 4 × 4 × 3 Monkhorst–Pack *k*-point grids at a spacing of 0.03 Å^−1^. The energy convergence accuracy is chosen at 1.0 × 10^−6^ eV, and the interaction force between atoms is kept below 0.01 eV/Å. The conjugate-gradient algorithm is utilized to relax all ions into their instantaneous ground state.

**Figure 1:**
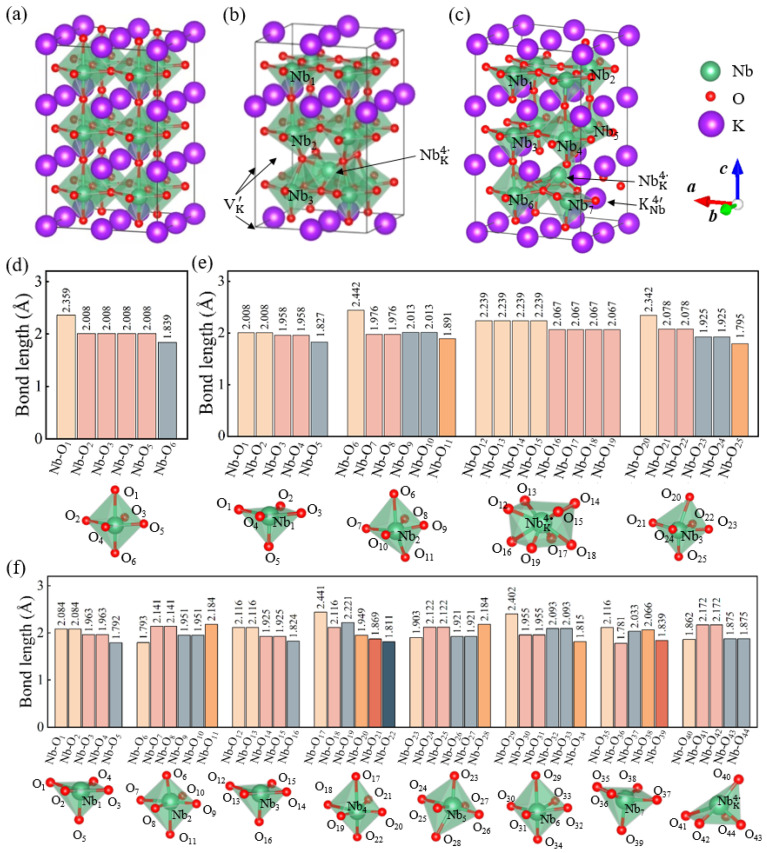
Optimized supercell structures and the corresponding lengths of Nb–O bonds for (**a**,**d**) KNbO_3_, (**b**,**e**) ${\mathrm{Nb}}_{K}^{4\cdot }$-$4V_{K}^{\prime }$-KNbO_3_, and (**c**,**f**) ${\mathrm{Nb}}_{K}^{4\cdot }$-$K_{\mathrm{Nb}}^{4\prime }$-KNbO_3_ models, respectively.

To study antisite defect effects, the tetragonal structure of KNbO_3_ having space group P4mm (no. 99) with experimental lattice constants, i.e., the unit cell parameters of *L*_a_ = *L*_b_ = 3.996 Å and *L*_c_ = 4.063 Å, is used as the initial configuration. The 2 × 2 × 3 supercell of the tetragonal KNbO_3_ unit cells (60 atoms) is employed to construct the antisite defect models, and their optimized atomic configurations are shown in Figure 1. In this work, we mainly pay attention to the excess Nb antisite defect, which introduces extra +4 charges to the model system. To balance charge and enhance structural stability, Nb antisites with A-site vacancies or antisite, namely (${\mathrm{Nb}}_{K}^{4\cdot }+4V_{K}^{\prime }$) and (${\mathrm{Nb}}_{K}^{4\cdot }+K_{\mathrm{Nb}}^{4\prime }$), are possible combinations of defect complexes in KNbO_3_. For (${\mathrm{Nb}}_{K}^{4\cdot }+4V_{K}^{\prime }$), the ${\mathrm{Nb}}_{K}^{4\cdot }$ is compensated by four K vacancies. In this scenario, a K atom at the A-site is randomly replaced by a Nb atom, and another four K vacancies are introduced to generate KNbO_3_ with (${\mathrm{Nb}}_{K}^{4\cdot }+4V_{K}^{\prime }$) defect clusters, which is simply abbreviated as the ${\mathrm{Nb}}_{K}^{4\cdot }$-$4V_{K}^{\prime }$-KNbO_3_ model. For the second scenario, the ${\mathrm{Nb}}_{K}^{4\cdot }$ is directly compensated by a $K_{\mathrm{Nb}}^{4\prime }$ antisite pair defect such that an A-site K atom is randomly selected and swapped with a Nb atom at the B-site, giving rise to the ${\mathrm{Nb}}_{K}^{4\cdot }$-$K_{\mathrm{Nb}}^{4\prime }$-KNbO_3_ model. It is noted that different antisite structures could be built according to the locations of these point defects. Hence, only the structure with the lowest total energy after geometry optimization is considered for the two antisite defect complex models in Figure 1b,c.

Additionally, the density functional perturbation theory (DFPT) method is employed to calculate and extract elastic and piezoelectric properties as the second derivatives of the total internal energy [43,44] as:

(1) $$C_{ij}=-\frac{\partial^{2}E}{\partial \eta_{i}\partial \eta_{j}}{|}_{\varepsilon }$$

(2) $$e_{\alpha j}=-\frac{\partial^{2}E}{\partial \eta_{i}\partial \eta_{j}}$$

where *C_ij_* is the elastic stiffness tensor with *i*,*j* = {1, 2, 3…6}, η is the homogeneous strain, *E* is the internal total energy, ε is the homogeneous electric field, and $e_{\alpha j}$ is the piezoelectric stress tensor with α = {1, 2, 3}.

## 3. Results and Discussions

### 3.1. Optimized Structure and Formation Energy

The supercell parameters of the optimized tetragonal KNbO_3_ and its derivatives with the antisite defect complexes are summarized in Table 1. After geometric optimization, the stable structures of KNbO_3_ and ${\mathrm{Nb}}_{K}^{4\cdot }$-$4V_{K}^{\prime }$-KNbO_3_ can maintain the tetragonal phase, while the ${\mathrm{Nb}}_{K}^{4\cdot }$-$K_{\mathrm{Nb}}^{4\prime }$-KNbO_3_ model evolves into a triclinic phase. Among the constructed defects, both ${\mathrm{Nb}}_{K}^{4\cdot }$ and $V_{K}^{\prime }$ exhibit fourfold symmetry around the *c* axis, and hence the combination of ${\mathrm{Nb}}_{K}^{4\cdot }$ and $V_{K}^{\prime }$ could retain the tetragonal structure in ${\mathrm{Nb}}_{K}^{4\cdot }$-$4V_{K}^{\prime }$-KNbO_3_. As revealed in Table 1, the relaxed cell lengths of KNbO_3_ are *L*_a_ = *L*_b_ = 7.989 Å and *L*_c_ = 12.595 Å, which are in excellent agreement with the experimental values and previous DFT results [45,46], confirming that our computational methods are reasonable and the calculated results are reliable. Compared to KNbO_3_, the introduction of the ${\mathrm{Nb}}_{K}^{4\cdot }$ and $V_{K}^{\prime }$ defects has a negligible effect on the cell length *L*_c_, but a slight shrinkage in *L*_a_ by ~1.7% and volume by ~3.4%, ascribed to the K vacancies, whereas the antisite pair defects ${\mathrm{Nb}}_{K}^{4\cdot }$ and $K_{\mathrm{Nb}}^{4\prime }$ produce a larger expansion of *L*_c_ by ~5.7% and volume by ~6.4%, further resulting in the tetragonal-to-triclinic phase transition.

It is also interesting to note that, among several possible configurations, both the ${\mathrm{Nb}}_{K}^{4\cdot }$-$4V_{K}^{\prime }$-KNbO_3_ and ${\mathrm{Nb}}_{K}^{4\cdot }$-$K_{\mathrm{Nb}}^{4\prime }$-KNbO_3_ models with antisites and vacancy defects located on nearest-neighbor sites possess the lowest energy and thus the most stable atomic structure (see Figure 1 and Tables S1 and S2), indicating that the Nb antisite defect tends to bind strongly to either K vacancies or K antisite defect, which is in good accordance with the DFT predictions for AgNbO_3_ with antisite pair defects ${\mathrm{Nb}}_{\mathrm{Ag}}^{4\cdot }$ and ${\mathrm{Ag}}_{\mathrm{Nb}}^{4\prime }$ [47]. For the pristine KNbO_3_, the Nb cation is octahedrally coordinated with the O anions forming the corner-shared NbO_6_ octahedron, which contains three types of Nb–O bonds (one of them four, and the other two with one each in number) in Figure 1d. The local off-center displacement of Nb cations relative to O anions is revealed by the difference in the Nb–O bond lengths along the *c* axis (i.e., Δ = 0.52 Å) and is regarded as the intrinsic origin of the ferroelectricity in tetragonal KNbO_3_. However, in addition to the typical coordination number of 6 for Nb, the two different antisite defect complexes result in NbO_5_ and NbO_8_ groups with coordination numbers of 5 and 8, respectively, as revealed in Figure 1e,f. The bond lengths of Nb–O bonds formed by the Nb and surrounding O atoms in the three different environments are also shown in Figure 1d–f, where the cutoff of Nb–O bond length is determined from the bond valence parameter method [48,49,50] and is chosen as ~2.453 Å. It is clearly observed that the oxygen polyhedron presents significant distortion and tilting after incorporating the two antisite defect clusters, which in turn plays a vital role in electronic and electrical properties of KNbO_3_ crystals. Although the tetragonal phase structure is still maintained in ${\mathrm{Nb}}_{K}^{4\cdot }$-$4V_{K}^{\prime }$-KNbO_3_, the in-plane anisotropy is introduced and the out-of-plane bond length variations become larger as compared to the pristine KNbO_3_. This is mainly attributed to the lacking K–O bonds as well as a stronger coupling between Nb and O than that of K and O, thereby leading to the department of the nearest O from $V_{K}^{\prime }$ and the remarkable downward movement of ${\mathrm{Nb}}_{K}^{4\cdot }$, which is accompanied by the distortion of oxygen polyhedra. For ${\mathrm{Nb}}_{K}^{4\cdot }$-$K_{\mathrm{Nb}}^{4\prime }$-KNbO_3_, the structural asymmetry together with local heterogeneity is further intensified to compensate for the loss of the incipient NbO_6_ and KO_12_ groups induced by the inversion of neighboring Nb and K cations. Moreover, for the two antisite-defect KNbO_3_ models, atomic relaxations in the vicinity of the defects are likely caused, and the positions of the antisite defects, especially ${\mathrm{Nb}}_{K}^{4\cdot }$, are displaced substantially along the $\left[00\bar{1}\right]$ and/or [$\bar{1}\bar{1}0$] directions, giving rise to local dipoles and polarization, thus potentially boosting ferroelectric and piezoelectric performance.

To examine the relative difficulty of incorporating the two antisite defect complexes into the lattice, the formation energy of the charge-neutral (${\mathrm{Nb}}_{K}^{4\cdot }+4V_{K}^{\prime }$) and (${\mathrm{Nb}}_{K}^{4\cdot }+K_{\mathrm{Nb}}^{4\prime }$) clusters can be calculated as:

(3) $$E_{F}\left[{\mathrm{Nb}}_{K}^{4\cdot }+4V_{K}^{\prime }\right]=E_{t}^{d}\left[K_{n-5}{\mathrm{Nb}}_{n+1}O_{3n}\right]-E_{t}^{0}\left[K_{n}{\mathrm{Nb}}_{n}O_{3n}\right]+5\mu_{K}-\mu_{\mathrm{Nb}}$$

(4) $$E_{F}\left[{\mathrm{Nb}}_{K}^{4\cdot }+K_{\mathrm{Nb}}^{4\prime }\right]=E_{t}^{d}\left[K_{n}{\mathrm{Nb}}_{n}O_{3n}\right]-E_{t}^{0}\left[K_{n}{\mathrm{Nb}}_{n}O_{3n}\right]$$

where *E_F_* denotes the defect formation energy, $E_{t}^{d}$ ($E_{t}^{0}$) is the total energy of the supercell with (without) the defects, $\mu_{X}$ is the atomic chemical potential of the element X, and *n* is the number of formula units included in the supercell. Generally, the smaller the formation energy, the easier it is for antisite defect clusters to enter the lattice position. Consequently, as seen in Table 2, $E_{F}$ is 10.01 and 3.71 eV for (${\mathrm{Nb}}_{K}^{4\cdot }+4V_{K}^{\prime }$) and (${\mathrm{Nb}}_{K}^{4\cdot }+K_{\mathrm{Nb}}^{4\prime }$), respectively, signifying that the antisite defect cluster formation is non-spontaneous, and more importantly, the (${\mathrm{Nb}}_{K}^{4\cdot }+K_{\mathrm{Nb}}^{4\prime }$) pair defects are much easier to form than (${\mathrm{Nb}}_{K}^{4\cdot }+4V_{K}^{\prime }$). This is mainly attributed to the much greater energy cost required for losing four K cations at the same time than for of replacing only one Nb by K in KNbO_3_ crystals.

### 3.2. Electronic Properties

#### 3.2.1. Electron Localization Function

**Figure 2:**
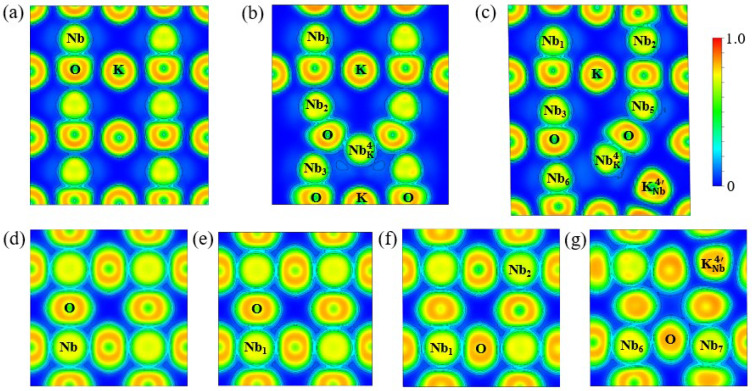
Two-dimensional electron localization function (ELF) profiles in the (110) plane for (**a**) KNbO_3_, (**b**) ${\mathrm{Nb}}_{K}^{4\cdot }$-$4V_{K}^{\prime }$-KNbO_3_, and (**c**) ${\mathrm{Nb}}_{K}^{4\cdot }$-$K_{\mathrm{Nb}}^{4\prime }$-KNbO_3_, and the ELF profiles in the (001) plane for (**d**) KNbO_3_, (**e**) ${\mathrm{Nb}}_{K}^{4\cdot }$-$4V_{K}^{\prime }$-KNbO_3_, and (**f**,**g**) ${\mathrm{Nb}}_{K}^{4\cdot }$-$K_{\mathrm{Nb}}^{4\prime }$-KNbO_3_. The marked Nb atoms refer to the ones in Figure 1b,c.

The electron localization function (ELF) is employed to analyze variation in the local chemical bonding environment of the pristine and antisite-defect KNO_3_, as shown in Figure 2. After replacing the A-site cation in Figure 2b, the Nb cation tightly bounds to eight O anions (four each at the top and bottom) and its center shifts downward remarkably, leading to a local deformed but yet symmetrical chemical bonding environment in the (110) plane. In addition to ${\mathrm{Nb}}_{K}^{4\cdot }$, the surrounding vacant four K atoms help aggravate the twisting and displacement of first-nearest-neighboring O atoms, which finally induces the noticeable distortion of the NbO_6_ octahedron. The position of the adjacent Nb cation (i.e., Nb_3_) is also slightly shifted outward due to the alike charge repulsion of ${\mathrm{Nb}}_{K}^{4\cdot }$. However, the B-site (001) plane far away from ${\mathrm{Nb}}_{K}^{4\cdot }$ displays a highly uniform and similar chemical bonding environment to the pristine KNbO_3_, as seen in Figure 2d,e. In contrast, for the antisite pair defects in Figure 2c, both the ${\mathrm{Nb}}_{K}^{4\cdot }$ and $K_{\mathrm{Nb}}^{4\prime }$ antisites are displaced significantly along the $\left[00\bar{1}\right]$ and [$\bar{1}\bar{1}0$] directions, resulting in asymmetrically structural and chemical bonding changes around the defect cluster. This undoubtedly contributes to the tetragonal-to-triclinic phase transition for ${\mathrm{Nb}}_{K}^{4\cdot }$-$K_{\mathrm{Nb}}^{4\prime }$-KNbO_3_. More intriguingly, the antisite pair defects produce more electron accumulation on Nb and O atoms on B-site planes (see Figure 2f,g), as compared to KNbO_3_ and ${\mathrm{Nb}}_{K}^{4\cdot }$-$4V_{K}^{\prime }$-KNbO_3_. Overall, the distinction of chemical bonding in various sites suggests that the influence of the antisite defect complexes is localized, which is a positive factor in enhancing ferroelectric and piezoelectric properties for defected KNbO_3_.

#### 3.2.2. Band Structure and Density of States

**Figure 3:**
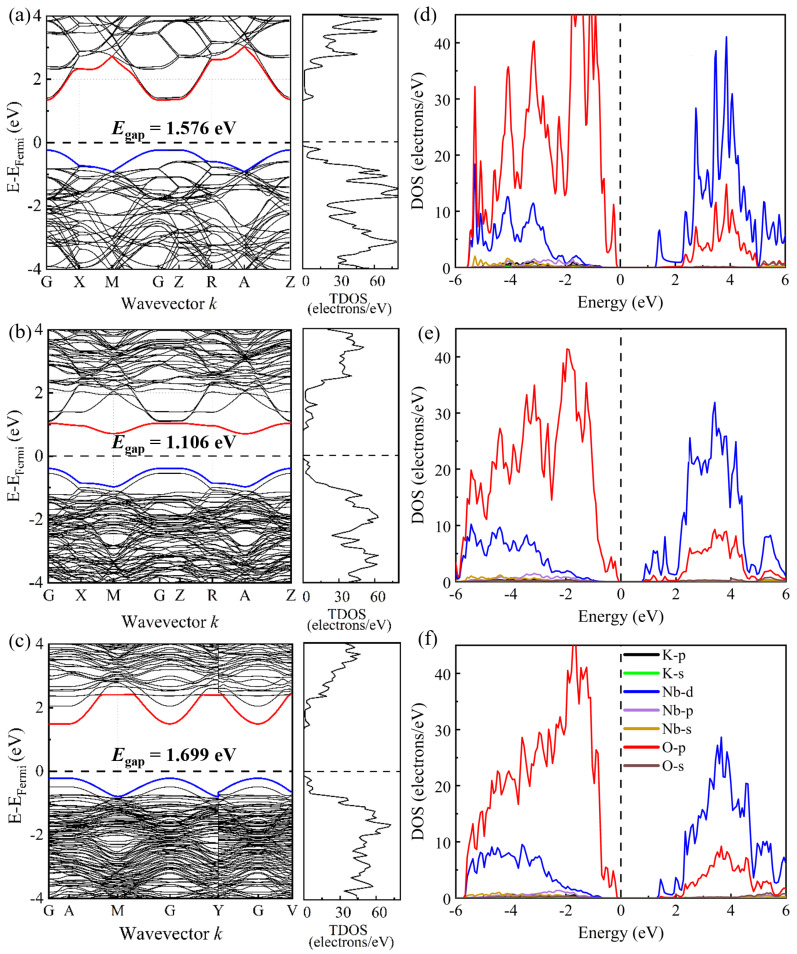
Energy band structures and the total and partial density of states (DOS) for different elements and orbitals in (**a**,**d**) KNbO_3_, (**b**,**e**) ${\mathrm{Nb}}_{K}^{4\cdot }$-$4V_{K}^{\prime }$-KNbO_3_, and (**c**,**f**) ${\mathrm{Nb}}_{K}^{4\cdot }$-$K_{\mathrm{Nb}}^{4\prime }$-KNbO_3_. The black dashed lines are the Fermi level, set to zero energy. The valance band maximum and the conduction band minimum are depicted by the blue and red lines, respectively, in (**a**–**c**).

The band structures along the symmetry directions as well as the total and partial electronic density of states (DOS) are further shown in Figure 3. Figure 3a shows that the pristine KNbO_3_ possesses an indirect band gap of ~1.576 eV, as valence band maxima (VBM) and conduction band minima (CBM) lie on different symmetry points, i.e., Z and G, of Brillouin zones. However, the calculated band gap is underestimated compared with the experimental value of 3.08 eV [51]. Although it is a common feature to undervalue band gaps due to the discontinuity in the exchange-correlation potentials [52], the DFT calculations are expected to be reliable and useful in examining relative changes in electronic band structure and energy band gaps for different KNbO_3_ models. As seen in Figure 3b,c, the calculated indirect band gaps for ${\mathrm{Nb}}_{K}^{4\cdot }$-$4V_{K}^{\prime }$-KNbO_3_ and ${\mathrm{Nb}}_{K}^{4\cdot }$-$K_{\mathrm{Nb}}^{4\prime }$-KNbO_3_ models are 1.106 and 1.699 eV, respectively, with VBM and CBM located around the G and A points. Compared to the pristine KNbO_3_, the conduction band moves downward (upward) to the Fermi level for ${\mathrm{Nb}}_{K}^{4\cdot }$-$4V_{K}^{\prime }$-KNbO_3_ (${\mathrm{Nb}}_{K}^{4\cdot }$-$K_{\mathrm{Nb}}^{4\prime }$-KNbO_3_), which causes the decrease (increase) in the band gap. Also, the conduction and valence bands in the antisite-defect models become denser, indicating that the electron localization intensifies and could improve the local polarization. To understand the variations in the band structure in detail, the partial DOS for different atoms near the Fermi level, i.e., the energy region from −6 to 6 eV, are presented in Figure 3d–f. It is clearly shown that the formation of the upper valance band from −6 to 0 eV for the three models is strongly influenced by O-2p states, followed by Nb-4d states, whereas K-states remain almost neutral. In the lower conduction band, above the Fermi level between 0 and 6 eV, the 4d-states of Nb along with partial 2p-states of O play a major role. The hybridization between Nb-4d and O-2p orbitals is thus dominant for the band gaps. As compared to the pristine KNbO_3_, the weaker p-d hybridization induced by the four K vacancies narrows the band gap for ${\mathrm{Nb}}_{K}^{4\cdot }$-$4V_{K}^{\prime }$-KNbO_3_, whereas the antisite pair defects strengthen p-d covalent interactions, leading to an increasing band gap for ${\mathrm{Nb}}_{K}^{4\cdot }$-$K_{\mathrm{Nb}}^{4\prime }$-KNbO_3_.

### 3.3. Ferro-Piezoelectric Properties

#### 3.3.1. Spontaneous Polarization

**Figure 4:**
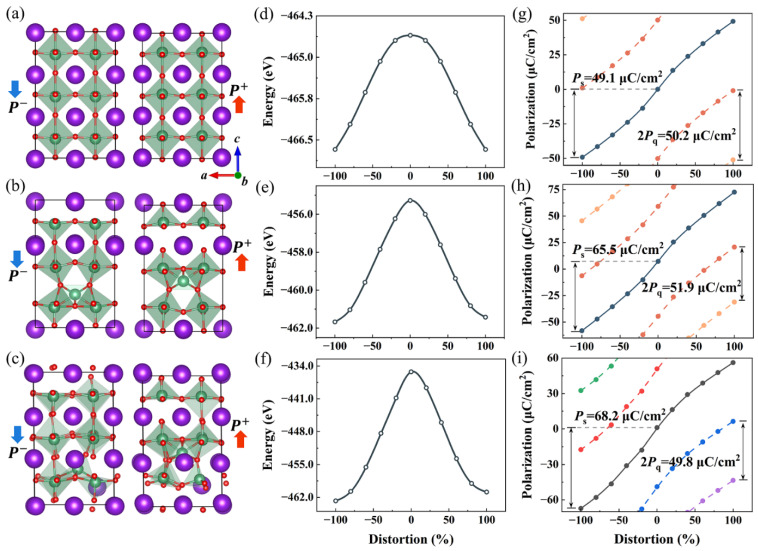
Atomic structures of pathways corresponding to the polarization switching of (**a**) KNbO_3_, (**b**) ${\mathrm{Nb}}_{K}^{4\cdot }$-$4V_{K}^{\prime }$-KNbO_3_, and (**c**) ${\mathrm{Nb}}_{K}^{4\cdot }$-$K_{\mathrm{Nb}}^{4\prime }$-KNbO_3_, and their corresponding energy (**d**–**f**) and polarization (**g**–**i**) profiles as functions of percentage distortion from the high symmetry (0% distortion) structure to the ferroelectric phase (±100% distortion) structure using the linear interpolation method. In (**g**–**i**), *P*_s_ is the spontaneous polarization along the −*c* axis and *P*_q_ is the polarization quantum. The open and solid dots are calculated points and the solid and dashed lines illustrate the evolution along the energy gradient and branches of the polarization lattice, respectively. Notice that the different colored lines represent the various branches of the lattice.

In modern theory of polarization, spontaneous polarization *P*_s_ is defined as the difference in polarization between the polar structure of interest and the non-polar (high-symmetry) reference structure. However, for the antisite-defect KNbO_3_, especially ${\mathrm{Nb}}_{K}^{4\cdot }$-$K_{\mathrm{Nb}}^{4\prime }$-KNbO_3_, the high-symmetry reference structure cannot be simply determined by inspection. Therefore, linear interpolation is employed to generate intermediate structures of two opposite polar states (*P*^−^ and *P*^+^), as seen in Figure 4a–c. The direction of polarization along the *c* axis is defined by the direction of the dipole moment of the oxygen polyhedron. If the Nb cation moves upward relative to the O anion, then it is in the *P*^+^ configuration; consequently, the *P*^−^ configuration is oppositely directed and negative. Note that the *P*^−^ configurations are consistent with the ones in Figure 1a–c. By taking the *P*^−^ and *P*^+^ configurations as the start and end points, respectively, the energy as a function of relative off-center displacement of Nb cations for the intermediate structures is shown in Figure 4d–f. As expected, the peak point approximately corresponds to a high-symmetry reference structure that can be utilized to calculate atomic displacement. The migration energy barrier *E*_a_ for direct *P*^−^ to *P*^+^ polarization switching is obtained as 2.1, 6.4, and 27.5 eV for the three models (see Table 3). The larger energy barrier for ${\mathrm{Nb}}_{K}^{4\cdot }$-$K_{\mathrm{Nb}}^{4\prime }$-KNbO_3_ is probably due to the local structural asymmetry with aggravated distortion and rotation of the oxygen polyhedron (see Figure 1f). Using the Berry phase method [53], the detailed identification of polarization changes is also illustrated in Figure 4g–i. The polarization branches are equally separated by 2*P*_q_, namely, *P*_q_ = *eR*/*V*, where *P*_q_ is the polarization quantum, *R* is the supercell lattice vector, and *V* is the volume. For tetragonal KNbO_3_, the magnitude of the spontaneous polarization *P*_s_ is estimated as 49.1 μC/cm^2^, which matches well with the experimental (37 μC/cm^2^ [54]) and previously reported theoretical values (45 μC/cm^2^ [55] and 51 μC/cm^2^ [56]). More intriguingly, the calculated *P*_s_ for ${\mathrm{Nb}}_{K}^{4\cdot }$-$4V_{K}^{\prime }$-KNbO_3_ and ${\mathrm{Nb}}_{K}^{4\cdot }$-$K_{\mathrm{Nb}}^{4\prime }$-KNbO_3_ are 65.5 and 68.2 μC/cm^2^, respectively, considerably larger than that of the pristine KNbO_3_.

To further elucidate the intrinsic origin of the improved polarization induced by antisite defect complexes, the *P*_s_ (see Table 3) is also estimated from the Born effective charge tensor Z^*^ and the corresponding atomic displacement δu of all atoms and is computed as:

(5) $$P_{s}=\frac{e}{V}\sum_{i,j}Z_{i,3j}^{*}\delta u_{i,j}$$

where *i* denotes the *i*th atom, and *j* has {1,2,3} options, corresponding to the *a*-, *b*-, and *c*-axis, respectively. In this way, the specific atomic polarization $P_{s}^{*}$ can be analyzed by gathering all of the *i* indices across the same type of atoms in Equation (5), as summarized in Table 4. It is clearly shown that the Nb cations contribute most to *P*_s_ for KNbO_3_ (64%) and ${\mathrm{Nb}}_{K}^{4\cdot }$-$4V_{K}^{\prime }$-KNbO_3_ (73%) due to their relatively larger $Z_{33}^{*}$ compared to K and O atoms, while for ${\mathrm{Nb}}_{K}^{4\cdot }$-$K_{\mathrm{Nb}}^{4\prime }$-KNbO_3_, both Nb and O play a major role in *P*_s_, with $P_{s}^{*}/P_{s}$ of 46% and 48%, respectively. In particular, one more Nb (i.e., ${\mathrm{Nb}}_{K}^{4\cdot }$) is incorporated in ${\mathrm{Nb}}_{K}^{4\cdot }$-$4V_{K}^{\prime }$-KNbO_3_, giving rise to additional $P_{s}^{*}$ of 10 μC/cm^2^ with a considerably high $\delta u_{3}$ of 0.87 Å, which is responsible for the improved *P*_s_. On the other hand, despite antisite defects, ${\mathrm{Nb}}_{K}^{4\cdot }$ and $K_{\mathrm{Nb}}^{4\prime }$ produce more $P_{s}^{*}$ of 9.35μC/cm^2^, and the total polarization contribution from Nb and K is comparable to that of KNbO_3_. Consequently, the overall largest *P*_s_ in ${\mathrm{Nb}}_{K}^{4\cdot }$-$K_{\mathrm{Nb}}^{4\prime }$-KNbO_3_ is dominated by the induced dipole moments of O anions in the surrounding cells rather than by the dipole moments of the defects themselves. The enhanced ferroelectricity resulting from the large off-centering of the antisite defects is thus accompanied by geometric asymmetry and severe distortion of oxygen polyhedron polarizing the regions surrounding the antisite pair defects.

#### 3.3.2. Piezoelectric Constants

The elastic stiffness tensor $C_{ij}$ and piezoelectric stress tensor $e_{\alpha j}$ predicted by using the DFPT method for different KNbO_3_ models are shown in Table 5 and Table 6. The piezoelectric strain tensor $d_{\alpha j}$ is also calculated and is related to $e_{\alpha j}$ and $C_{ij}$ as:

(6) $$d_{\alpha j}=\sum_{i=1}^{6}e_{\alpha i}S_{ij}$$

where $S_{ij}$ is the elastic compliance tensor and is the inverse matrix of the elastic stiffness tensor $C_{ij}$.

For tetragonal KNbO_3_ and ${\mathrm{Nb}}_{K}^{4\cdot }$-$4V_{K}^{\prime }$-KNbO_3_, *d*_15_, *d*_31_, and *d*_33_ are the independent piezoelectric strain constants, while for the triclinic ${\mathrm{Nb}}_{K}^{4\cdot }$-$K_{\mathrm{Nb}}^{4\prime }$-KNbO_3_, all components are independent (see Equation (S12) in the Supplementary Document) and only 3 of them are summarized for a better illustration. Since our KNbO_3_ model in Figure 1a presents the *P*_s_ along the –*c* axis due to the downward displacements of Nb cations relative to the O anions, the calculated piezoelectric constants might have the opposite signs as compared with other work. As revealed in Table 5 and Table 6, the elastic stiffness constants and piezoelectric constants of KNbO_3_ show an overall excellent agreement with previous computational and experimental results [56,57,58,59,61]. Especially, *d*_33_ is the most important piezoelectric constant, responding to a uniaxial stress parallel to the spontaneous polarization direction, while the *d*_31_ component is smaller than *d*_33_ since they correspond to the uniaxial stress in a direction orthogonal to the strong Nb–O bonds. This variation can be further confirmed by the considerably smaller *S*_31_ than *S*_33_ (see Equation (S2)). However, the relatively small *e*_15_ and *S*_55_ result in the smallest *d*_15_, signifying that a miniature change in the in-plane polarization (i.e., *P*_1_) is anticipated when a shear stress $\sigma_{13}$ is applied. More intriguingly, as compared to the pristine KNbO_3_, the defected KNbO_3_ models exhibit significantly larger piezoelectric strain tensor, which is related to their weakened elastic stiffness. In particular, the ${\mathrm{Nb}}_{K}^{4\cdot }$-$K_{\mathrm{Nb}}^{4\prime }$-KNbO_3_ presents the *d*_33_ around three times higher than that of KNbO_3_. The incorporation of the antisite pair defects breaks the symmetry of the threefold-degenerate Nb–O bonds and thus effectively stretches the oxygen polyhedron along the *c* axis due to the amplified *S*_33_ (Equation (S10)). This undoubtedly generates a much larger change in the polarization parallel to the existing spontaneous dipole, *P*_3_, when subjected to a uniaxial stress $\sigma_{33}$. Also, the ${\mathrm{Nb}}_{K}^{4\cdot }$-$K_{\mathrm{Nb}}^{4\prime }$-KNbO_3_ model exhibits relatively large *d*_13_ and *d*_23_, as associated with the in-plane polarization *P*_1_ and *P*_2_ in the presence of $\sigma_{33}$, which arises primarily from the large *e*_11_ and *e*_22_, respectively, as shown in Equations (S11) and (S12). Hence, it is reasonably assumed that the antisite defect complexes, in particular (${\mathrm{Nb}}_{K}^{4\cdot }+K_{\mathrm{Nb}}^{4\prime }$), aid in improving the local structural and chemical heterogeneity, eventually promoting ferroelectric and piezoelectric performances.

## 4. Conclusions

In this work, the structural, electronic, ferroelectric, and piezoelectric properties of the antisite-defect KNbO_3_ single crystals have been examined using DFT calculations. As compared to tetragonal ${\mathrm{Nb}}_{K}^{4\cdot }$-$4V_{K}^{\prime }$-KNbO_3_, (${\mathrm{Nb}}_{K}^{4\cdot }+K_{\mathrm{Nb}}^{4\prime }$) pair defects can enter the lattice of KNbO_3_ with a smaller defect formation energy and induce considerable distortion of the Nb-O polyhedron, leading to a tetragonal-to-triclinic phase transition. Meanwhile, the ELF profiles show asymmetrically structural and chemical bonding changes around ${\mathrm{Nb}}_{K}^{4\cdot }$ and $K_{\mathrm{Nb}}^{4\prime }$ defects, and their band structures reveal an increased band gap beneficial to the enhancement of breakdown resistance. Moreover, the effect of the antisite defect complex on ferroelectric polarization and switching pathway was unveiled by means of the Berry phase method. Both the (${\mathrm{Nb}}_{K}^{4\cdot }+4V_{K}^{\prime }$) and (${\mathrm{Nb}}_{K}^{4\cdot }+K_{\mathrm{Nb}}^{4\prime }$) clusters generate additional ferroelectric polarization, even with (${\mathrm{Nb}}_{K}^{4\cdot }+K_{\mathrm{Nb}}^{4\prime }$) producing a more pronounced spontaneous polarization and higher migration barrier of polarization reversal than (${\mathrm{Nb}}_{K}^{4\cdot }+4V_{K}^{\prime }$). By analyzing the atomic polarization contribution based on Born effective charge tensor and atomic displacement, the improved ferroelectricity in ${\mathrm{Nb}}_{K}^{4\cdot }$-$K_{\mathrm{Nb}}^{4\prime }$-KNbO_3_ is dominated by geometric asymmetry and severe distortion of Nb-O polyhedron polarizing the regions surrounding the antisite pair defects. Furthermore, the piezoelectric strain tensor was estimated, and the presence of ${\mathrm{Nb}}_{K}^{4\cdot }$ and $K_{\mathrm{Nb}}^{4\prime }$ pair defects could result in a *d*_33_ about two and three times larger than those of the ${\mathrm{Nb}}_{K}^{4\cdot }$-$4V_{K}^{\prime }$-KNbO_3_ and pristine KNbO_3_, respectively, due to a moderate *e*_33_ and a significantly higher elastic compliance constant *S*_33_. Overall, the antisite defect complexes, in particular (${\mathrm{Nb}}_{K}^{4\cdot }+K_{\mathrm{Nb}}^{4\prime }$), help improve the local structural and chemical heterogeneity, and subsequently, polarization and atomic displacement responses under macroscopic stress, thereby boosting the ferro-piezoelectric performance of KNbO_3_ single crystals. The findings in this work therefore provide a fundamental understanding and intuitive guidelines for designing high-performance lead-free ferroelectric perovskites by exploring the greatest potential of intrinsic antisite defect complexes as well as their critical roles in boosting ferroelectric coupling and electromechanical response.

## Figures and Tables

**Table 1:** ***L*_a_**, ***L*_b_** and ***L*_c_** as cell lengths (Å), ***α***, ***β*** and ***γ*** as angles (°), volume (Å^3^), energy (eV) and space group for different KNbO_3_.

Model	*L*_a_, *L*_b_	*L*_c_	*α*	*β*	*γ*	Volume	Energy	Space Group
KNbO_3_	7.989	12.595	90.00	90.00	90.00	803.8	−466.70	P4mm
${\mathrm{Nb}}_{K}^{4\cdot }$-$4V_{K}^{\prime }$-KNbO_3_	7.854	12.584	90.00	90.00	90.00	776.2	−461.67	P4mm
${\mathrm{Nb}}_{K}^{4\cdot }$-$K_{\mathrm{Nb}}^{4\prime }$-KNbO_3_	8.014	13.317	89.40	89.40	90.25	855.2	−462.99	P1

**Table 2:** Defect-forming energy $E_{F}$ (eV) and element chemical potential $\mu_{X}$ (eV).

Model	$E_{F}$	Element	$\mu_{X}$
${\mathrm{Nb}}_{K}^{4\cdot }$-4$V_{K}^{\prime }$-KNbO_3_	10.01	Nb	−10.202
${\mathrm{Nb}}_{K}^{4\cdot }$-$K_{\mathrm{Nb}}^{4\prime }$-KNbO_3_	3.71	K	−1.043

**Table 3:** Migration energy barrier *E*_a_ (eV) and spontaneous polarizations along the −*c* axis *P*_s_^a^ and *P*_s_^b^ (μC/cm^2^) using the Berry phase method and Born effective charge, respectively.

Model	*E*_a_	*P*_s_^a^	*P*_s_^b^
KNbO_3_	2.1	49.1	39.2
${\mathrm{Nb}}_{K}^{4\cdot }$-$4V_{K}^{\prime }$-KNbO_3_	6.4	65.5	55.1
${\mathrm{Nb}}_{K}^{4\cdot }$-$K_{\mathrm{Nb}}^{4\prime }$-KNbO_3_	27.5	68.2	57.0

**Table 4:** Averaged Born effective charge Z^*^, atomic displacement δ*u* (Å), the effective atomic polarization along the −*c* axis $P_{s}^{*}$ (μC/cm^2^), and its corresponding percentage contribution $P_{s}^{*}/P_{s}$ for different types of atoms in the pristine and defected KNbO_3_ models. The oxygen atoms are divided into four types: O^Nb^ and O^K^, representing the nearest-neighboring O atoms located around anti-site defects ${\mathrm{Nb}}_{K}^{4\cdot }$ and $K_{\mathrm{Nb}}^{4\prime }$; and O^I^ (collinear with Nb along the *c* axis) and O^II^ (coplanar with the Nb in the *ab* plane), highlighting the structural difference between the two O sites.

Model	Element	$Z_{31}^{*}$	$Z_{32}^{*}$	$Z_{33}^{*}$	$\delta u_{1}$	$\delta u_{2}$	$\delta u_{3}$	$P_{s}^{*}$	$P_{s}^{*}/P_{s}$
KNbO_3_	K	0	0	1.23	0	0	−0.10	2.85	7%
	Nb	0	0	5.99	0	0	−0.18	25.16	64%
	O^I^	0	0	−4.59	0	0	0.08	9.17	24%
	O^II^	0	0	−1.31	0	0	0.03	1.99	5%
${\mathrm{Nb}}_{K}^{4\cdot }$-$4V_{K}^{\prime }$-KNbO_3_	K	0	0	1.33	0	0	−0.20	3.78	7%
	Nb	0	0	5.25	0	0	−0.22	30.25	55%
	${\mathrm{Nb}}_{K}^{4\cdot }$	0	0	5.54	0	0	−0.87	9.96	18%
	O^Nb^	0	0	−2.80	0	0	0.05	2.51	5%
	O^I^	0	0	−3.98	0	0	0.11	7.29	13%
	O^II^	0	0	−1.18	0	0	0.02	1.32	2%
${\mathrm{Nb}}_{K}^{4\cdot }$-$K_{\mathrm{Nb}}^{4\prime }$-KNbO_3_	K	0	0	1.27	0	0	−0.10	2.68	5%
	$K_{\mathrm{Nb}}^{4\prime }$	−0.04	−0.04	1.33	−0.18	−0.18	−0.21	0.50	1%
	Nb	−0.15	−0.15	5.08	−0.02	−0.02	−0.17	17.41	31%
	${\mathrm{Nb}}_{K}^{4\cdot }$	−0.65	−0.65	5.17	−0.20	−0.20	−0.97	8.85	15%
	O^K^	−0.22	−0.22	−1.58	0.04	0.04	0.25	2.79	5%
	O^Nb^	0.41	0.41	−1.61	−0.10	−0.10	0.36	7.65	13%
	O^I^	0.03	0.03	−3.87	0.02	0.02	0.20	14.42	25%
	O^II^	0.06	0.06	−1.21	0.04	0.04	0.06	2.69	5%

**Table 5:** Elastic stiffness constants (GPa) for different KNbO_3_ models.

Model	*C*_11_	*C*_12_	*C*_13_	*C*_22_	*C*_33_	*C*_44_	*C*_55_	*C*_66_
KNbO_3_	328.90	82.54	67.13		75.69	78.89		90.28
KNbO_3_ cal. [57]	340.5	82.1	66.9		89.5	78.5		91.7
KNbO_3_ cal. [58]	325.39	83.36	66.10		78.12	74.86		85.12
KNbO_3_ cal. [59]	324.90	82.67	66.85		67.97	30.67		89.59
${\mathrm{Nb}}_{K}^{4\cdot }$-$4V_{K}^{\prime }$-KNbO_3_	132.74	237.46	58.91		44.13	50.59		69.43
${\mathrm{Nb}}_{K}^{4\cdot }$-$K_{\mathrm{Nb}}^{4\prime }$-KNbO_3_	130.40	105.84	57.94	131.55	34.96	20.57	17.48	63.66

**Table 6:** Piezoelectric constants (C/m^2^ for *e_ij_* and pC/N for *d_ij_*) for different KNbO_3_ models.

Model	*e*_15_	*e*_31_	*e*_33_	*d*_15_	*d*_31_	*d*_33_
KNbO_3_	−0.32	0.066	−3.58	−4.11	11.09	−66.97, 80 a
KNbO_3_ cal. [59]	2.05 b	−0.066 b	3.70 b	26.12	−8.77	54.40
KNbO_3_ cal. [60]	−1.31 b	−0.24 b	4.08 b	−15.90	−7.42	39.89
${\mathrm{Nb}}_{K}^{4\cdot }$-$4V_{K}^{'}$-KNbO_3_	−1.86	−0.31	−3.02	−36.83	18.22	−116.29
${\mathrm{Nb}}_{K}^{4\cdot }$-$K_{\mathrm{Nb}}^{4\text{'}}$-KNbO_3_	−0.70	0.001	−1.52	86.60	54.22	−228.27

^a^ Experimental result for tetragonal KNbO_3_ [61]. ^b^ The calculated piezoelectric stress constants *e_ij_* are derived based on *C_ij_* and *d_ij_* from each reference.

## Data Availability

The original contributions presented in the study are included in the article/Supplementary Materials, further inquiries can be directed to the corresponding authors.

## Supplementary Materials

The following supporting information can be downloaded at: https://www.mdpi.com/article/10.3390/ma17143442/s1, Table S1: Constructed possible models and corresponding total energies for ${\mathrm{Nb}}_{K}^{4\cdot }$-$4V_{K}^{\prime }$-KNbO_3_; Table S2: Constructed possible models and corresponding total energies for ${\mathrm{Nb}}_{K}^{4\cdot }$-$K_{\mathrm{Nb}}^{4\prime }$-KNbO_3_; Equations (S1)–(S12): Elastic stiffness matrix (GPa), elastic flexibility matrix (10^−3^ GPa^−1^), piezoelectric stress matrix (C/m^2^), and piezoelectric strain matrix (pC/N) for KNbO_3_, ${\mathrm{Nb}}_{K}^{4\cdot }$-4$V_{K}^{\prime }$-KNbO_3_ and ${\mathrm{Nb}}_{K}^{4\cdot }$-$K_{\mathrm{Nb}}^{4\prime }$-KNbO_3_.
