# Supplementary material for: Insights into Antisite Defect Complex Induced High Ferro-Piezoelectric Properties in KNbO3 Perovskite: First-Principles Study
Source: Materials (Basel). 2024 Jul 11;17(14):3442. doi: 10.3390/ma17143442 (PMC11277845; doi:10.3390/ma17143442)
Supplement: Supplementary file 1 [file materials-17-03442-s001.zip › materials-3064844-supplementary.pdf]

Insights into Antisite Defect Complex Induced High Ferro-Piezoelectric Properties in $KNbO_3$ Perovskite: First-Principles Study

Bei Li[a,b,*], Yilun Zhang[a,b], Meng Wang[a,b], Xu Zhang[a,b,*], Xiaofeng Zhangc, Kai Liu[a,*]

[a] School of Materials Science and Engineering, Wuhan University of Technology, Wuhan 430070, China

[b] Research Center for Materials Genome Engineering, Wuhan University of Technology, Wuhan 430070, China

[c] National Engineering Laboratory for Modern Materials Surface Engineering Technology & The Key Lab of Guangdong for Modern Surface Engineering Technology, Institute of New Materials, Guangdong Academy of Science, Guangzhou 510650, China

* Corresponding authors. zhxu@ whut.edu.cn; liukai19870222@163.com

Table S1 Constructed possible models and corresponding total energies for $Nb_K^{4\cdot}$-$4V_K'$-$KNbO_3$

Model	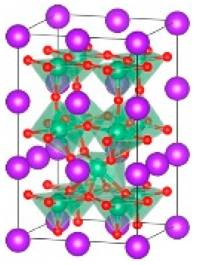	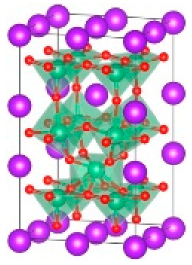	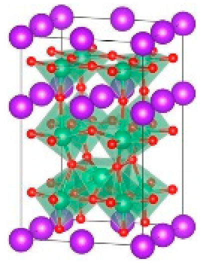	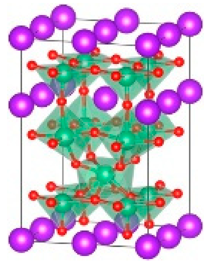
Energy/eV	-461.063	-461.114	-461.357	-460.854
Model	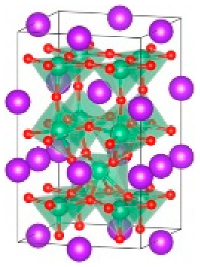	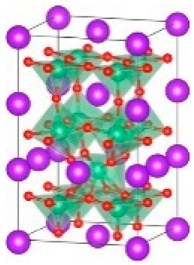	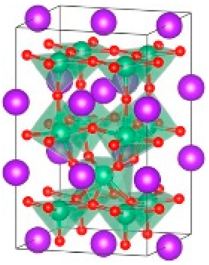	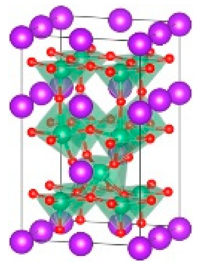
Energy/eV	-460.992	-461.053	-461.061	-461.171
Model	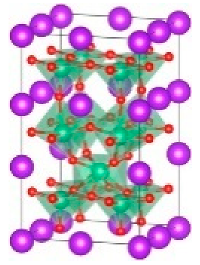	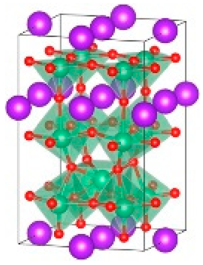	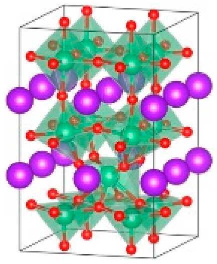	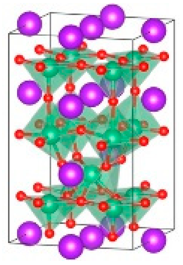
Energy/eV	-460.901	-461.674	-461.265	-461.472

Table S2 Constructed possible models and corresponding total energies for $Nb_{K}^{4\cdot}$-$K_{Nb}^{4\prime}$-$KNbO_3$

Model	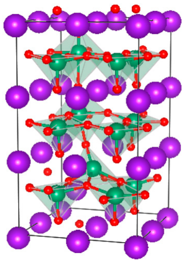	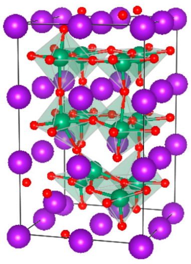	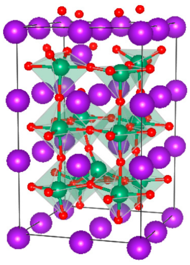	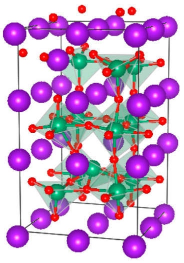
Energy/eV	-462.987	-462.408	-460.681	-460.677

Elastic stiffness matrix (GPa), elastic flexibility matrix (10^{-3} GPa^{-1}), piezoelectric stress matrix (C/m^2), and piezoelectric strain matrix (pC/N) for pristine $KNbO_3$:

$$C_{ij} = \begin{bmatrix} 328.90 & 82.54 & 67.12 & 0 & 0 & 0 \\ 82.54 & 328.90 & 67.12 & 0 & 0 & 0 \\ 67.12 & 67.12 & 75.69 & 0 & 0 & 0 \\ 0 & 0 & 0 & 78.89 & 0 & 0 \\ 0 & 0 & 0 & 0 & 78.89 & 0 \\ 0 & 0 & 0 & 0 & 0 & 90.28 \end{bmatrix} \quad (S1)$$

$$S_{ij} = \begin{bmatrix} 3.74 & -0.32 & -3.03 & 0 & 0 & 0 \\ -0.32 & 3.74 & -3.03 & 0 & 0 & 0 \\ -3.03 & -3.03 & 18.59 & 0 & 0 & 0 \\ 0 & 0 & 0 & 12.68 & 0 & 0 \\ 0 & 0 & 0 & 0 & 12.68 & 0 \\ 0 & 0 & 0 & 0 & 0 & 11.08 \end{bmatrix} \quad (S2)$$

$$e_{ij} = \begin{bmatrix} 0 & 0 & 0 & 0 & -0.33 & 0 \\ 0 & 0 & 0 & -0.33 & 0 & 0 \\ 0.07 & 0.07 & -3.58 & 0 & 0 & 0 \end{bmatrix} \quad (S3)$$

$$d_{ij} = \begin{bmatrix} 0 & 0 & -0.02 & 0 & -4.11 & 0 \\ 0 & 0.02 & -0.06 & -4.21 & 0 & 0 \\ 11.09 & 11.09 & -66.97 & 0.05 & 0 & 0 \end{bmatrix} \quad (S4)$$

Elastic stiffness matrix (GPa), elastic flexibility matrix (10^{-3} GPa^{-1}), piezoelectric stress matrix (C/m^2), and piezoelectric strain matrix (pC/N) for $Nb_K^{4\bullet}$-$4V_K'$-$KNbO_3$:

$$C_{ij} = \begin{bmatrix} 132.74 & 237.46 & 58.91 & 0 & 0 & 0 \\ 237.46 & 134.41 & 59.93 & 0 & 0 & 0 \\ 58.91 & 59.93 & 44.13 & 0 & 0 & 0 \\ 0 & 0 & 0 & 50.59 & 0 & 0 \\ 0 & 0 & 0 & 0 & 50.57 & 0 \\ 0 & 0 & 0 & 0 & 0 & 69.43 \end{bmatrix} \quad (S5)$$

$$S_{ij} = \begin{bmatrix} -2.42 & 7.18 & -6.53 & -0.07 & 0.10 & 0.01 \\ 7.18 & -2.47 & -6.24 & 0.09 & -0.07 & -0.01 \\ -6.53 & -6.24 & 39.84 & -0.04 & -0.04 & -0.01 \\ -0.07 & 0.09 & -0.04 & 19.77 & 0.00 & 0 \\ 0.10 & -0.07 & -0.04 & 0 & 19.77 & 0 \\ 0.01 & -0.01 & -0.01 & 0 & 0.00 & 14.40 \end{bmatrix} \quad (S6)$$

$$e_{ij} = \begin{bmatrix} 0.02 & 0 & 0.02 & 0 & -1.86 & 0 \\ -0.01 & 0.02 & 0.01 & -1.86 & 0 & 0 \\ -0.31 & -0.31 & -3.02 & 0 & 0 & 0 \end{bmatrix} \quad (S7)$$

$$d_{ij} = \begin{bmatrix} -0.34 & 0.21 & 0.53 & 0.01 & -36.83 & 0 \\ 0.24 & -0.32 & 0.32 & -36.79 & -0.04 & -0.06 \\ 18.22 & 17.36 & -116.29 & 0.10 & 0.10 & 0.02 \end{bmatrix} \quad (S8)$$

Elastic stiffness matrix (GPa), elastic flexibility matrix (10^{-3} GPa^{-1}), piezoelectric stress matrix (C/m^2), and piezoelectric strain matrix (pC/N) for $Nb_K^{4\bullet}$-$K_{Nb}^{4\prime}$-$KNbO_3$:

$$C_{ij} = \begin{bmatrix} 130.40 & 105.84 & 57.94 & 8.08 & -24.62 & 2.18 \\ 105.84 & 131.55 & 57.89 & -22.38 & 7.87 & 2.00 \\ 57.94 & 57.89 & 34.96 & -3.81 & -3.68 & 0.69 \\ 8.08 & -22.88 & -3.81 & 20.57 & 27.48 & 1.01 \\ -24.62 & 7.87 & -3.68 & 27.48 & 17.48 & 1.23 \\ 2.18 & 2.00 & 0.69 & 1.01 & 1.23 & 63.66 \end{bmatrix} \tag{S9}$$

$$S_{ij} = \begin{bmatrix} 14.02 & 7.69 & -35.83 & 13.90 & -13.07 & -0.30 \\ 7.69 & 15.32 & -37.90 & -11.25 & 13.68 & -0.42 \\ -35.83 & -37.90 & 150.66 & -1.94 & 1.28 & 0.80 \\ 13.90 & -11.25 & -1.94 & 1.92 & 21.24 & -0.54 \\ -13.07 & 13.68 & 1.28 & 21.24 & -0.47 & -0.33 \\ -0.30 & -0.42 & 0.80 & -0.54 & -0.33 & 15.74 \end{bmatrix} \tag{S10}$$

$$e_{ij} = \begin{bmatrix} -5.09 & 1.38 & 0.26 & 0.03 & -0.70 & 0.03 \\ 1.38 & -5.07 & 0.26 & -0.68 & 0.03 & 0.02 \\ 0 & 0 & -1.52 & -0.09 & -0.09 & 0.07 \end{bmatrix} \tag{S11}$$

$$d_{ij} = \begin{bmatrix} -60.54 & -37.70 & 168.24 & -101.46 & 86.60 & 1.80 \\ -38.95 & -69.01 & 183.76 & 75.08 & -101.68 & 2.59 \\ 54.22 & 57.21 & -228.27 & 0.82 & -3.86 & 0.04 \end{bmatrix} \tag{S12}$$
